# Two-Stage Deep Neural Network *via* Ensemble Learning for Melanoma Classification

**DOI:** 10.3389/fbioe.2021.758495

**Published:** 2022-01-18

**Authors:** Jiaqi Ding, Jie Song, Jiawei Li, Jijun Tang, Fei Guo

**Affiliations:** ^1^ School of Computer Science and Technology, College of Intelligence and Computing, Tianjin University, Tianjin, China; ^2^ Shenzhen Institute of Advanced Technology, Chinese Academy of Sciences, Shenzhen, China; ^3^ School of Computer Science and Engineering, Central South University, Changsha, China

**Keywords:** melanoma classification, ensemble learning, deep convolutional neural network, image segmentation, dermoscopy images

## Abstract

Melanoma is a skin disease with a high fatality rate. Early diagnosis of melanoma can effectively increase the survival rate of patients. There are three types of dermoscopy images, malignant melanoma, benign nevis, and seborrheic keratosis, so using dermoscopy images to classify melanoma is an indispensable task in diagnosis. However, early melanoma classification works can only use the low-level information of images, so the melanoma cannot be classified efficiently; the recent deep learning methods mainly depend on a single network, although it can extract high-level features, the poor scale and type of the features limited the results of the classification. Therefore, we need an automatic classification method for melanoma, which can make full use of the rich and deep feature information of images for classification. In this study, we propose an ensemble method that can integrate different types of classification networks for melanoma classification. Specifically, we first use U-net to segment the lesion area of images to generate a lesion mask, thus resize images to focus on the lesion; then, we use five excellent classification models to classify dermoscopy images, and adding squeeze-excitation block (SE block) to models to emphasize the more informative features; finally, we use our proposed new ensemble network to integrate five different classification results. The experimental results prove the validity of our results. We test our method on the ISIC 2017 challenge dataset and obtain excellent results on multiple metrics; especially, we get 0.909 on accuracy. Our classification framework can provide an efficient and accurate way for melanoma classification using dermoscopy images, laying the foundation for early diagnosis and later treatment of melanoma.

## 1 Introduction

Skin cancer is a major public health problem, with more than 5 million new cases diagnosed annually in the United States (Siegel et al., 2016; Codella et al., 2018). Melanoma is the fastest-growing and deadliest form of skin cancer in the world; it causes many deaths each year. However, it is noticed that melanoma multiplies more slowly in the early stages, so if it is diagnosed early and treated promptly, the survival rates of patients can be greatly improved.

Pigmentation lesions occur on the skin surface, and dermoscopic technology was introduced to improve the diagnosis of skin melanoma. Dermoscopy is a non-invasive skin imaging technique that can magnify and illuminate skin areas, and then enhance visualization of deep skin by eliminating surface reflections. Compared with standard photography, dermoscopy images can greatly improve the accuracy of diagnosis (Kittler et al., 2002; Codella et al., 2018). Dermatologists usually use “ABCD” rule to evaluate skin lesions (Stolz, 1994; Moura et al., 2019). This rule analyzes asymmetry, boundary irregularities, color variations, and structures of lesions (Xie et al., 2016). However, the differentiation of skin lesions by dermatologists from dermoscopy images is often time consuming and subjective, and the diagnostic accuracy depends largely on the professional level, so inexperienced dermatologists may not be able to make accurate judgments. Therefore, we urgently need an automatic recognition method that is non-subjective and can assist dermatologists to make more accurate diagnosis.

**FIGURE 1:**
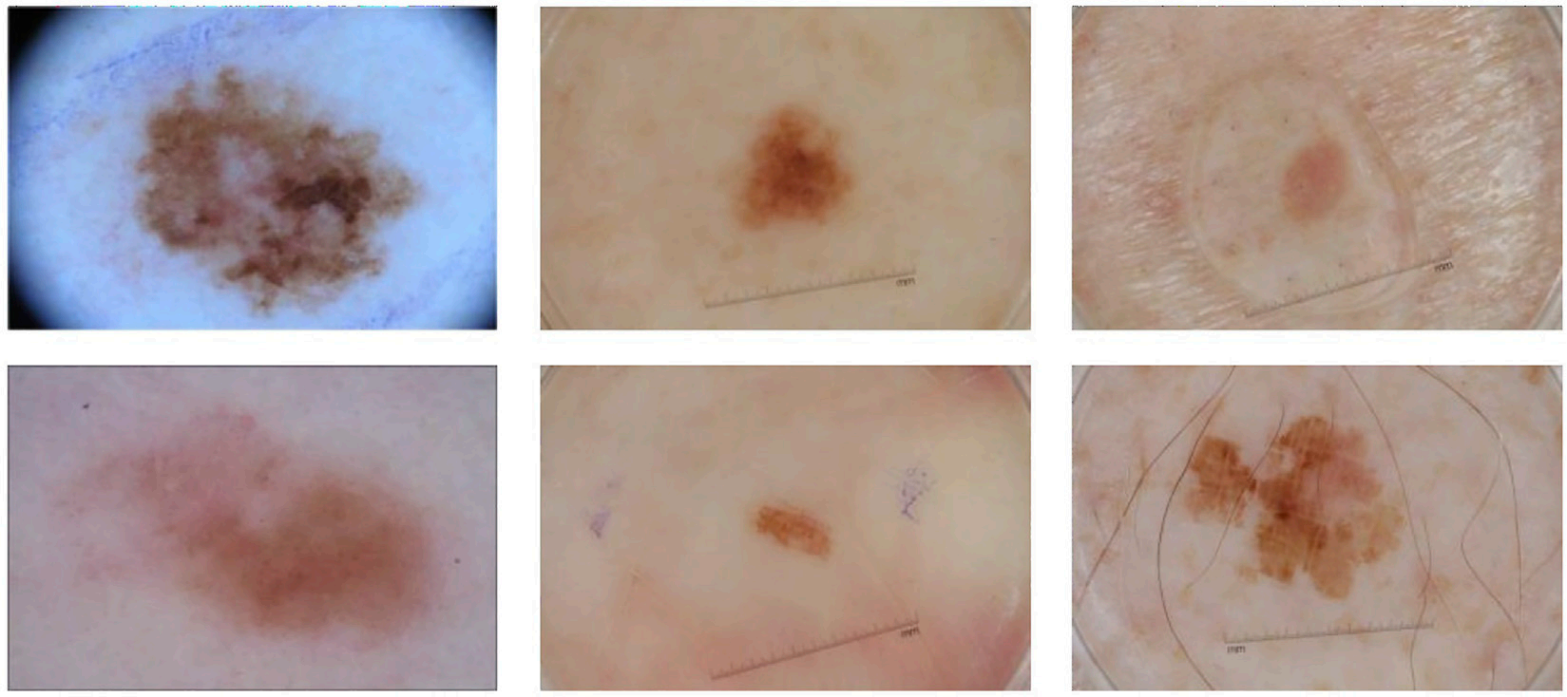
Some samples of dermoscopy images. From left to right: malignant melanoma, benign nevis, and seborrheic keratosis.

However, there are still many challenges in automated recognition of melanoma, we show them in Figure 1. The first column of Figure 1 shows malignant melanoma, the second column shows benign nevis, and the third column shows seborrheic keratosis. First, skin lesions have great inter-class similarity and intra-class variation in color, shape, and texture; the different classes of skin lesion have high visual similarity. Second, the area of skin lesions in dermoscopy images varies greatly, and the boundaries between skin lesions and normal skin are blurred in some images. Third, artifacts such as hair, rulers, and texture in dermoscopy images may make it hard to identify melanoma changes. All these factors make automatic recognition more difficult.

To solve these problems, many researches have made attempts. Generally, automatic analysis models include four steps: image preprocessing, border detection or segmentation, feature extraction, and classification. In early works, a large number of studies used shallow models to classify dermoscopy images, mainly using low-level features such as shape, color, texture, or their combination (Ganster et al., 2001; Mishra and Celebi, 2016); however, these shallow models for extracting low-level features lack high-level representation and powerful generalization capabilities. In recent years, convolutional neural network has made great breakthroughs in image analysis tasks (Krizhevsky et al., 2012; He et al., 2015; Long et al., 2015; Shin et al., 2016; Chen et al., 2017), especially the deep convolutional neural networks (DCNNs), which can extract deep features and have better discrimination ability, have achieved improved performance. So researchers started to apply DCNN to analyze medical images (Roychowdhury et al., 2015; Myronenko, 2018), including image-based melanoma classification. However, deep neural networks still face great challenges in the field of medical image analysis. DCNN requires large datasets to obtain more effective features, while medical image data are often difficult to obtain and the datasets are relatively small. If a small dataset is used directly for deep network training, it will lead to over-fitting of the model. Moreover, a single network may not be able to extract all the informative features, and it is actually difficult to train a model that performs well in all aspects. Therefore, we propose an integrated model based on transfer learning to combine the results of multiple models to get better performance.

In this paper, we propose a novel two-stage ensemble method based on deep convolutional neural networks. In the first stage, we perform the image segmentation, we use a segmentation network to generate lesion segmentation masks, and then we use these masks to resize the original images so that they are the same size. In the second stage, we implement image classification, we utilize five state-of-the-art networks to extract features, and we add Squeeze-and-Excitation Blocks (Hu et al., 2018) to the network to help emphasize more informative features. Then we construct a new neural network using local connection to integrate the classification results of these models, so that we can obtain the final classification result. We evaluate our method on ISIC 2017 challenge dataset and obtain the best results on some metrics.

## 2 Related Works

### 2.1 Traditional Methods

Traditional methods are usually based on manually extracted features to classify dermoscopy images, including features of color and texture. The “ABCD” rule is the standard used by dermatologists, and there are many automatic classification methods that are based on this rule. Barata et al. (2013) introduced two different dermoscopy image detection systems; one used a global approach to classify skin lesions and the other used local features and a bag-of-features (BoF) classifier. Ganster et al. (2001) used manual features containing shape, boundary, and radiometric features to describe lesions, and then used KNN (K-Nearest Neighbor) to classify melanoma. Celebi et al. (2007) extracted descriptors related to shape, color, and texture from dermoscopy images and used non-linear support vector machines to classify melanoma lesions. Capdehourat et al. (2011) first preprocessed the image with hair removal, then used segmentation algorithm to segment each image, and finally trained the AdaBoost classifier with descriptors containing shape and color information.

### 2.2 Deep CNN Models

In recent years, convolutional neural network (CNN) has been widely used in image segmentation (Roychowdhury et al., 2015; Dai et al., 2016; Myronenko, 2018) and classification (Krizhevsky et al., 2012; Simonyan and Zisserman, 2014; Szegedy et al., 2015; He et al., 2016; Szegedy et al., 2016; Chollet, 2017; Szegedy et al., 2017; Huang et al., 2017), object detection (He et al., 2015; Liu et al., 2016; Redmon et al., 2016), and other scopes of computer vision (Xiao et al., 2021; Chen et al., 2021b). CNN models have multiple layers to extract features. The network extractor mainly has two parts, convolutional layers and pooling layers, and the network classifier is the fully connected layer. Convolutional layers use convolutional kernels to carry out convolution operation with input images to extract features. Kernels obtain features of the whole image by sliding on it as a window. Also, the convolution operation of each kernel is only connected to a local area called receptive field of the input. Receptive field and weight sharing are important parts of convolution neural network; they can effectively change the amount of training parameters. Pooling operation is a kind of down sampling; its purpose is to reduce the training time, increase the receptive field, and prevent over-fitting, including widely used max pooling and average pooling. In addition, the fully connected layer maps the learned feature representation to the label space for classification. If you need to classify the samples into *n* classes, there are *n* neurons in the last fully connected layer.

Many CNN models have great performance on computer vision tasks (Cao et al., 2021; Chen et al., 2021a; Feng et al., 2021). Studies have shown that increasing the number of layers in a network can significantly improve the performance (Simonyan and Zisserman, 2014; Szegedy et al., 2015). In recent years, deep CNN has been proposed and performed well in the field of dermoscopy recognition. Codella et al. (2015) used integrated CNN, sparse coding, and SVM for melanoma classification. Yu et al. (2016) proposed an automatic recognition method based on DCNN and residual learning, which first segmented skin lesions and identified melanoma with two classifiers. Yu et al. (2018) proposed a network based on DCNN and used feature coding strategy to generate representative features. Xie et al. (2016) processed the incomplete inclusion of lesions in dermoscopy images and proposed a new boundary feature that can describe boundary characteristics of complete and incomplete lesions. Lai and Deng (2018) combined the extracted low-level features (color, texture) with the extracted high-level features of the convolutional neural network for classification. González-Díaz (2019) proposed a CAD system called DermaKNet to help dermatologists in their diagnosis. DermaKNet was divided into four parts, first segmenting the lesions in the dermoscopic images using the Lesion Segmentation Network (LSN), then using the segmented masks to perform data augmentation on the original data, and next the Dermoscopic Structure Segmentation Network (DSSN) was used to segment the global and local features of the image; finally, the image classification is performed using the ResNet50-based network. Xie et al. (2020) proposed MB-DCNN to perform segmentation and classification of dermoscopic images. They first used a coarse segmentation network (coarse-SN) to generate a coarse lesion mask, which was used to assist the mask-guided classification network (mask-CN) to locate and classify lesions, and the localized lesion regions were fed into the enhanced segmentation network (enhanced-SN) to obtain a fine-grained lesion segmentation map. They also proposed a new rank loss to alleviate the sample class imbalance problem. Gessert et al. (2020) proposed a patch-based attention architecture to classify high-resolution dermoscopic images, which was able to provide global contextual information to improve the accuracy of classification. In addition, they proposed a new weighting loss to address the class imbalance in the data. Zunair and Hamza (2020) first performed conditional image synthesis by learning inter-class mapping and synthesizing samples of under-represented classes from over-represented classes using unpaired image-to-image translations, thereby exploiting inter-class variation in the data distribution. Then the set of these synthetic and original data was used to train a deep convolutional neural network for skin lesion classification. Bdair et al. (2021) proposed FedPerl, a semi-supervised federated learning approach, which used peer learning and ensemble averaging to build communities and encourage their members to learn from each other so that they can generate more accurate pseudo-labels. They also proposed the peer anonymization (PA) technique as a core component of FedPerl. Datta et al. (2021) explored the goal of Soft-Attention to emphasize the value of important features and to suppress features that cause noise. Then they compared the performance of VGG, ResNet, Inception ResNet v2, and DenseNet architectures for classifying skin lesions with and without the Soft-Attention mechanism. The results showed that the Soft-Attention mechanism improved the performance of the baseline networks.

## 3 MaterialS and Methods

**FIGURE 2:**
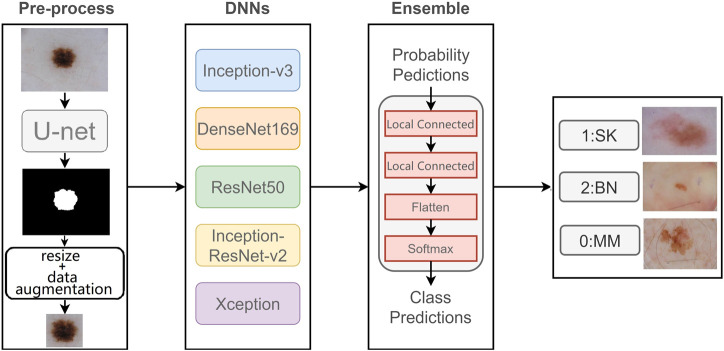
Flowchart of our proposed model.

In this section, we introduce our proposed two-stage ensemble network model. First, in the first stage, we train a segmentation network to segment skin lesions to get the lesion mask, and resize the mask area to generate lesion image with the same size. Then, in the second stage, we use five networks with good classification results on ImageNet to classify dermoscopy images, respectively. Also, we propose a new neural network to integrate the five results. The entire framework is shown in Figure 2.

### 3.1 Data Pre-Processing

The deep network model needs a large amount of training data to better fit the real data distribution, and the lack of training data may lead to over-fitting and other problems, which will seriously affect the classification ability of the model. However, most medical image datasets do not have much data, which is one of the biggest challenges of medical image analysis. Data augmentation is one of the common solutions to increase the amount of training data, and it can improve the model generalization ability. Therefore, we use different data augmentation methods on the original dataset, including rotation transform with 180°, flipping the images horizontally and vertically, and moving the image height and width direction by 10*%*, so that each original image generates five new samples.

### 3.2 Skin Lesion Segmentation

Lesion segmentation plays an important role in the automatic analysis of skin lesion. It can separate the lesion from the normal skin; therefore, the classifier can better identify the lesion features.

Unlike the classification network, which takes the images of fixed size as input and then outputs the class of each image, it gradually reduces the resolution of original images through convolution and max-pooling, and the feature maps it finally obtains are much smaller than the original image, then it classifies the feature maps through several fully connected layers. However, the output of segmentation network is the equal-sized prediction maps with input images. In the segmentation network, each pixel is a sample that needs to be classified into positive or negative. Therefore, the segmentation network needs decoder to compensate for the loss of feature resolution that is caused by max-pooling. In our experiment, we use deconvolution operation in the decoder to obtain a prediction mask with the same size as the input image.

U-net (Ronneberger et al., 2015) is an end-to-end deep convolutional neural network, which does not contain a fully connected layer, but is composed of convolution layers and up-sampling layers. U-net has an encoder and a decoder. Encoder reduces the dimension of images and extracts feature; it is composed of four blocks, each of which consists two 3 × 3 convolution layers followed by a ReLU activation function, and one max-pooling layer with stride of 2. Decoder also has four blocks, each containing a deconvolution layer, which double the size of feature maps, and two 3 × 3 convolution layers. So as for up-sampling operation in the decoder, U-net combines the output of up-sampling layer with feature map of symmetric encoder using skip-connection, so that the final output of network can consider both the shallow spatial information and deep semantic information. In this way, the outputs of the same size of the corresponding blocks in the encoder and decoder can be concatenated for segmentation and then the final prediction map is generated through a 1 × 1 convolution layer.

We train a U-net network to segment the original images and generate segmentation masks to show the lesion. These segmentation masks are used to crop the original images to help the classification network better focus on lesion features.

### 3.3 Skin Lesion Classification

The skin lesions have great inter-class similar visual effects; if we train our classification network to use the original images, the results will be less effective. So we divide our classification model into three stages. First, we segment skin lesions from original images using segmentation network and then resize them into a fixed size. Next, we use five classification networks with SE block to classify dermoscopy images. Finally, we construct a convolution neural network to ensemble five results.

### 3.4 Resize

The size of lesions varies greatly, and in most dermoscopy images, the lesion area only occupies a small part of the image, and most parts are non-lesion areas that may affect classification. In this case, if the original images are directly classified, the size of skin lesion will seriously affect the performance of network. Therefore, we first segment skin lesions from the dermoscopy images, then adjust the segmented lesion to a fixed size. Compared with the network trained on original dermoscopy images, the network trained on segmented and resized images can better extract features and has better performance.

### 3.5 SE Block

The features extracted by a convolutional neural network can directly affect the results of subsequent tasks, either segmentation or classification. Therefore, improving the quality of the feature representation of the network is crucial to improve the final classification results. The role of the Squeeze-and-Excitation block (Hu et al., 2018) is to further improve the classification accuracy by emphasizing the more important and informative features in the feature map. The SE block can be seen as a channel-wise attention mechanism, which emphasizes the importance of some features in the task by giving them greater weights. The specific strategy is shown in the next section that follows.

SE block is primarily concerned with the dependencies between feature channels. SE block does squeeze and excitation operation on feature maps U(*H* × *W* × *C*). The squeeze operation includes a global average pooling; it can map feature maps to feature vectors. The *c-th* feature map can be expressed as

$$z_{c}=F_{sq}\left(u_{c}\right)=\frac{1}{H\times W}\overset{H}{\underset{i=1}{\sum }}\overset{W}{\underset{j=1}{\sum }}u_{c}\left(i,j\right)$$ (1)

where H and W represent the height and width of feature map separately. Then the excitation operation includes two fully connected layers, a ReLU activation and sigmoid activation, so that it is able to fit complex correlations between channels by adding non-linear processing through dimensional changes. The formula can be expressed as

$$s=F_{ex}\left(z,W\right)=\sigma \left(W_{2}\delta \left(W_{1}z\right)\right)$$ (2)

where *δ* represents ReLU function and *σ* means Sigmoid, and *W*_1_ and *W*_2_ are the weights of the first and second fully connected layer separately. In this way, the values in this feature vector are mapped to 0, −, 1. Then the vector *s* can be multiplied as a channel descriptor with the original feature map to obtain the weighted feature map:

$$\tilde{x_{c}}=F_{scale}\left(u_{c},s_{c}\right)=s_{c}u_{c}$$ (3)

**FIGURE 3:**
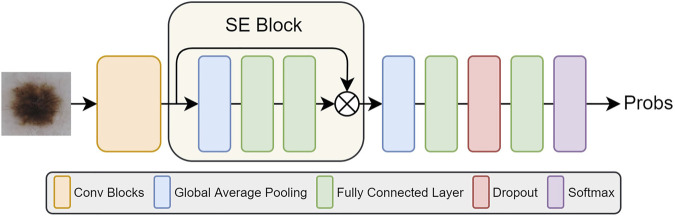
The illustration of five network structures after adding SE Blocks.

Therefore, SE block is used to standardize feature maps according to their importance and highlight more informative feature maps, thus it can improve the network performance effectively. The schematic of adding SE Block to the five networks is shown in Figure 3. We add the SE Block in the same position in each network, that is, after feature extraction (orange box in Figure 3) and before final classification of each network.

### 3.6 Network Model

For ensemble problems, in addition to the ensemble method, the basic model of integration is also important. We use five state-of-the-art networks as basic network for our integration, which are Inception-v3, Densenet169, ResNet50, Inception-ResNet-v2, and Xception. These networks all have good performance on image classification tasks.

#### 3.6.1 Inception-v3

Inception module (Szegedy et al., 2015) used 1 × 1, 3 × 3, and 5 × 5 convolution layers at the same time, then concatenated three kinds of outputs and transmitted it to the next module. In this way, it can consider information of different scales at the same time by increasing the width of the network. In addition, Inception module also can split channel-wise and spatial-wise correlation and small size of convolution kernel can greatly reduce the parameters. On the basis of Inception module, Inception-v3 (Szegedy et al. (2016)) replaced the 5 × 5 convolution layer in the original Inception network with two 3 × 3 convolution layers to further reduce the amount of parameters while maintaining the receptive field and increasing the ability of representation. Furthermore, another innovation of Inception-v3 was to decompose a large *n* × *n* convolution kernel (for example, a 7 × 7 convolution kernel) into two one-dimensional convolution kernels with the size of *n* × 1 and 1 × *n*, respectively. This can increase the model’s non-linear representation capability while reducing the risk of over-fitting.

#### 3.6.2 ResNet-50

ResNet (He et al., 2016) appeared to alleviate the problem of vanishing/exploding gradients. ResNet was composed of a set of residual blocks, each of which is composed of several layers, including convolutional layer, ReLU layer, and batch normalization layer. Also, for each residual block, its input was directly added to its output via identity, a short connection that allowed us to perform residual learning; this is the key to solve gradient problems when training deep networks. A residual block can be formulated as

$$H_{l}=H_{l-1}+F\left(H_{l-1}\right)$$ (4)

where *H*_*l*_ and *H*_*l*_ − 1 are the output and input of the *l-th* residual block, respectively. F(x) represents the residual mapping function of stacked layers. It is obvious that the dimensions of *H*_*l*_ − 1 and F(*H*_*l*_ − 1) should be equal. However, convolution operation usually changes the dimensions, so a linear projection *W*_*s*_ is used to match the dimensions. So Eq. 4 can be converted to

$$H_{l}=W_{s}H_{l-1}+F\left(H_{l-1}\right)$$ (5)

Therefore, ResNet-50 was obtained by stacking the residual blocks to make the final network layer count to 50.

#### 3.6.3 Densenet169

**FIGURE 4:**
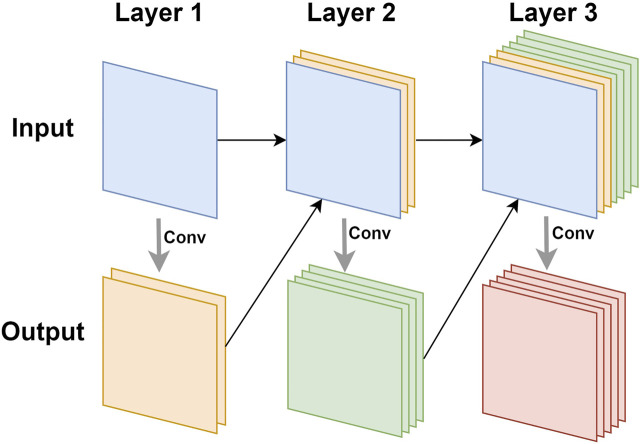
The illustration of feature reuse of dense block.

Densenet (Huang et al., 2017) was inspired by Resnet. It also used connections to alleviate the problem of vanishing gradients, but it did not use residual blocks to achieve this goal. Densenet was composed of dense blocks. In each dense block, as shown in Figure 4, the input of the *n-th* layer was the result of the concatenation of all the previous *n*−1 layers. In this way, when performing related operations on the *n-th* layer, the utilization of the features of all the previous layers can be maximized. This feature reuse method can make the features work better while reducing the amount of parameters.

#### 3.6.4 Inception-ResNet-v2

Inception-ResNet-v2 (Szegedy et al., 2017) combined Inception module with residual learning. It was based on Inception-v4, which was deeper and better than Inception-v3, but had more parameters. Inception-ResNet-v2 added residual identities to different types of Inception modules of Inception-v4, so that the network converged faster, and the training time of the network was shortened.

#### 3.6.5 Xception

Xception (Chollet, 2017) was an improvement to Inception-v3. It mainly replaced ordinary convolution in Inception-v3 with depthwise separable convolution. The multiple convolution kernels of depthwise separable convolution only processed part of feature maps produced by the previous layer. For example, for the result of 1 × 1 convolution output from the Inception module, depthwise separable convolution referred to using three 3 × 3 convolution kernels to operate on one-third of the channel of this result, and finally three results from three 3 × 3 convolution kernels were concatenated together. In this way, the amount of parameters can be greatly reduced. Also, the author believed that Xception can decouple the channel correlation and spatial correlation of the features, thereby producing better computational results.

We use these five pre-trained networks on ImageNet as feature extractors, then add SE blocks after every extractor to emphasize more informative features. Then, a full connected layer of 128-dimension is used to generate the final feature vector, and finally we use softmax classifier to obtain class predictions.

#### 3.6.6 Ensemble Learning

There are usually two ways to ensemble multiple networks: averaging and voting. Averaging refers to the average results of multiple networks, with each network accounting for the same proportion, so that they have the same influence on the final result. However, for each class, some networks produce better results, and some have relative worse effect; taking the average directly would reduce the advantage of good networks.

For voting ensemble, we can implement it through neural networks. In detail, the neural network we build for ensemble learning is equivalent to a new classifier, whose input is the classification probabilities from five networks, and whose output is the final classification result. The reason we chose to build the classifier with locally connected layer instead of fully connected layer is that fully connected layer will be connected to all the outputs of the previous layer, while locally connected layer will only be connected to parts of the previous layer. In this case, the part of the output of the ensemble network will only be determined by a specific input, and the prediction of one class will not be influenced by the other two classes because the local connection layer extracts features for each class separately, so the network will produce more accurate classification results. This new network is used to integrate the results of the five networks, consisting of two local connected layers and a softmax layer, as shown in Figure 2. The result has an improvement over the averaging ensemble method.

## 4 Results

### 4.1 Dataset

**FIGURE 5:**
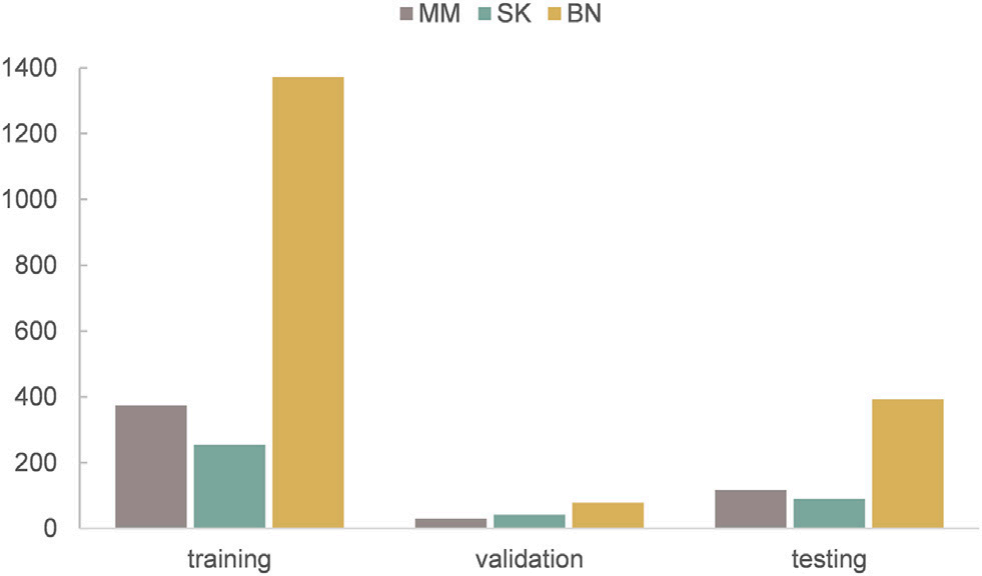
The distribution of training, validation, and test sets of ISIC 2017 challenge dataset.

The dataset we use to evaluate our method was provided by ISIC 2017 challenge organized by The International Society for Digital Imaging of the Skin (Codella et al., 2018). It includes 2,750 dermoscopy images and is divided into three subsets: 2,000 for training, 150 for validation, and 600 for testing. The images in the dataset are classified as three classes: benign nevi (BN), seborrheic keratosis (SK), or melanoma (MM). The details of ISIC 2017 challenge dataset is shown in Table 1, MM refers to melanoma, SK refers to seborrheic keratosis, and BN refers to benign nevi. Also, we can see from Figure 5 that the distribution of training, validation, and test sets is very uneven; the images of BN are far more than the images of the other two classes in three subsets. In addition, the ISIC 2017 dataset also provides dermoscopy images with their binary masks as their segmentation ground truth.

**TABLE 1:** Details of ISIC 2017 challenge dataset.

Subsets	MM	SK	BN	Total
Training	374	254	1,372	2,000
Validation	30	42	78	150
Testing	117	90	393	600

The ISIC 2017 challenge consists of two binary classification subtasks: melanoma or others and seborrheic keratosis or others.

### 4.2 Implementation

Our method is implemented with Keras on a computer with GeForce RTX 2080Ti GPU. The images with the size of 224 × 224 are taken as input of model, so all dermoscopy images are resized to 224 × 224 after segmentation. We use Adam algorithm as optimizer, and the learning rate is set as 0.0001 initially. Our epoch number is set to 100 initially. To prevent over-fitting, we use early stopping method with patience of 10 epochs.

### 4.3 Metrics

We use accuracy (ACC), recall, precision, F1-score, and AUC (area under ROC curve) as classification metrics. They are defined as

$$ACC=\frac{TP+TN}{TP+TN+FP+FN}$$ (6)

$$recall=\frac{TP}{TP+FN}$$ (7)

$$precision=\frac{TP}{TP+FP}$$ (8)

$$f1score=\frac{2\times precision\times recall}{precision+recall}$$ (9)

where TP, TN, FP, and FN denote the number of true positive, true negative, false positive, and false negative. The number of three classes in our dataset are imbalanced, so in this case, ACC cannot well reflect the performance of our classifier; therefore, we use AUC, the same indicator as ISIC classification challenge (Codella et al., 2018), as the main metric.

### 4.4 Performance on Multi-Class Classification

**FIGURE 6:**
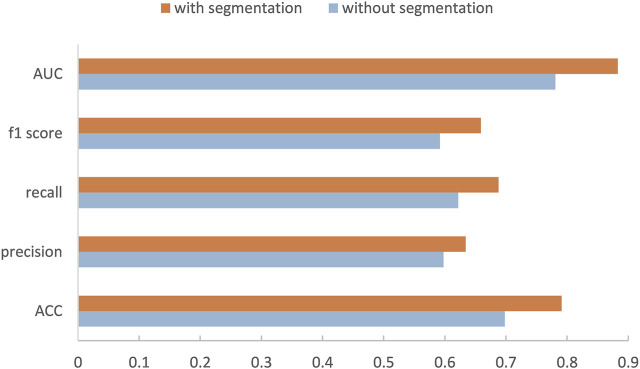
Performance of our method with or without segmentation.

Our method is divided into three parts. After segmenting and cropping the original dermoscopy images, five pre-trained models are used to do classification, and then the results of these models are ensembled to generate the final result. To verify our method, in this section, we modify the dataset and convert the two binary classification tasks into a multi-classification task. Then we compare the performance with and without segmentation and resize, and the performance before and after ensemble. Table 2 shows the experimental results with and without segmentation under one pre-trained network called Inception-v3. It can be seen that the network has better performance running on the segmented images than on the original images. As shown in Figure 6, especially on ACC and AUC, the results of network with segmentation get 0.791 and 0.883, respectively, which are much higher than that of network without segmentation. This is because the size of skin lesions varies greatly, and there are some interference factors such as artificial rulers in the original dermoscopy images. Segmentation can remove these interference factors to some extent, so that the network can better identify features.

**TABLE 2:** Classification results with or without segmentation.

Methods	ACC	Precision	Recall	f1 score	AUC
Without segmentation	0.698	0.598	0.622	0.592	0.781
With segmentation	0.791	0.634	0.688	0.659	0.883

In the ensemble stage, we construct a neural network model with two local connected layers with softmax classifier to fuse the results of five basic networks. Our new ensemble method can further improve the performance, and is better than the commonly used ensemble method. Table 3 lists the results of the five pre-trained models we use and the results of averaging ensemble and our ensemble method. (The bold numbers in the table of this article are the maximum values of their columns) It can be seen that the fusion model have better performance than any single network and average method on most metrics. For the recall and f1 scores, our ensemble method is 0.033 and 0.007 lower than Xception, but it is higher than other methods in other metrics. Especially, it has a 2*%* improvement on AUC over the result of best network, i.e., Xception. Also, our ensemble method is better than traditional average ensemble method on all metrics except for recall.

**TABLE 3:** Results of different networks and two ensemble methods on multi-classification task. (The bold numbers in the table of this article are the maximum values of their columns).

Methods	ACC	Precision	Recall	f1 score	AUC
Inception-v3	0.792	0.634	0.688	0.659	0.883
Densenet169	0.800	0.739	0.727	0.722	0.881
Resnet50	0.762	0.676	0.678	0.672	0.864
Inception-Resnet-v2	0.800	0.736	0.726	0.725	0.873
Xception	0.810	0.75	**0.748**	**0.748**	0.896
Average	0.793	0.724	0.724	0.719	0.880
Ensemble	**0.851**	**0.769**	0.715	0.741	**0.913**

We also compare the amount of parameters and training time of different networks (including our ensemble network). From Table 4, we can see that the classification networks have more parameters, especially Inception-Resnet-v2, which has up to 54.87 M. However, compared with these classification networks, our ensemble network has very few parameters, only 423. For training time, since the classification networks have been pre-trained on ImageNet, we just need to fine-tune the networks during training, and our training set is small, so we can see that the training time of each network is relatively short (when training 100 epochs). At the same time, we can also notice that the training time of the network is not entirely determined by their parameters, but is also related to the parallelism of the model and the memory access cost. In addition, these five classification networks are independent of each other, so they can be trained at the same time, which can also greatly reduce training time. Finally, our ensemble network requires very little training time, only 20 s.

**TABLE 4:** The amount of parameters and the training time of each network.

Networks	Inception-v3	Densenet169	Resnet50	Inception-resnet-v2	Xception	Ensemble
Params	22.56 M	13.22 M	24.32 M	54.87 M	21.59 M	423
Time(s)	1,900	3,200	1,900	3,000	2,700	20

### 4.5 Performance on Binary Classification

**FIGURE 7:**
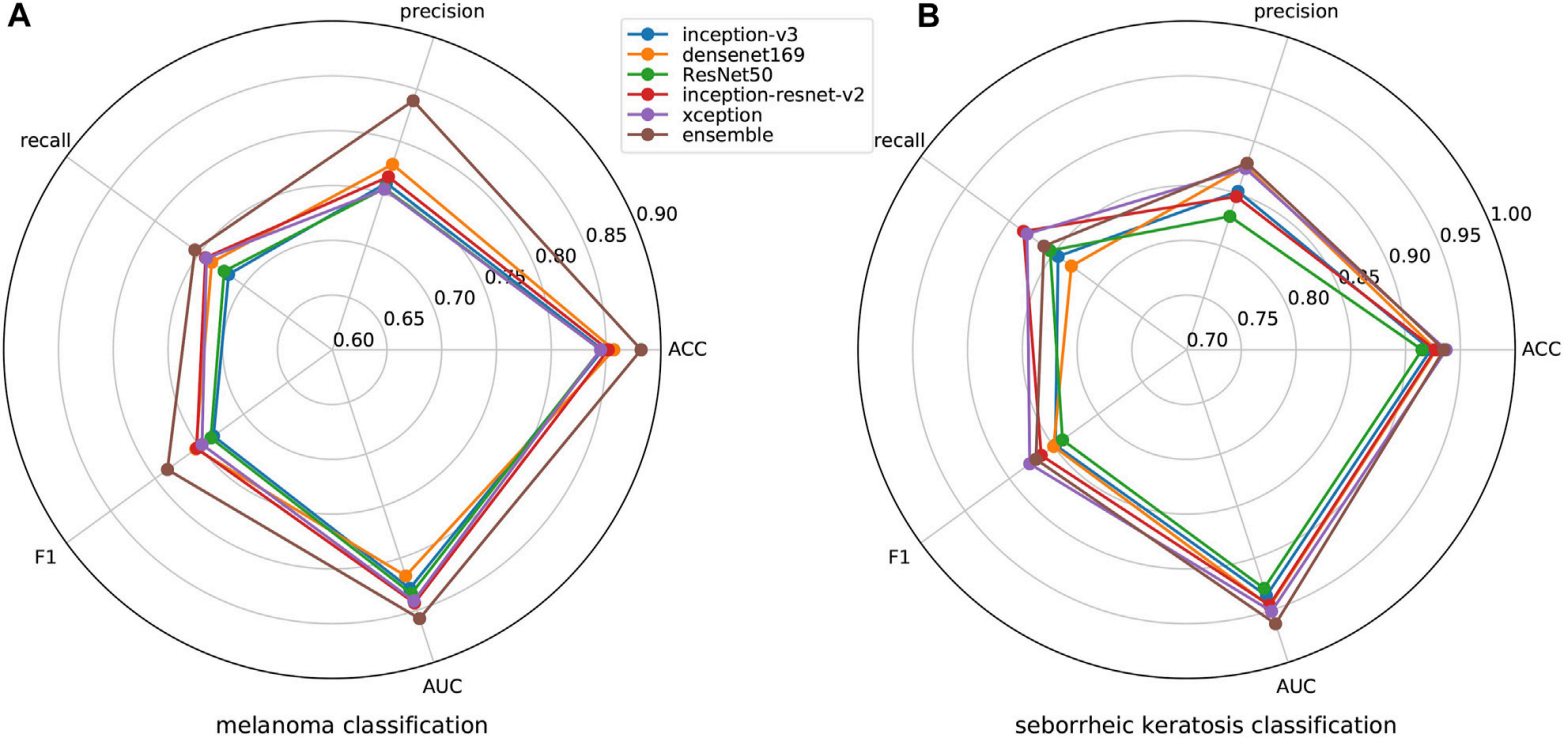
Results of melanoma and seborrheic keratosis classification for different networks.

ISIC 2017 challenge has two binary classification tasks, melanoma or others and seborrheic keratosis or others, so we also carry out the experiment regarding challenge tasks. We show the results of melanoma classification and seborrheic keratosis classification in the form of radar diagrams, as shown in Figure 7. Polar coordinates represent different metrics and each line represents a network. It can be seen that our method performs pretty well on both tasks. For the classification of melanoma, it is clear that our performance is the highest in all metrics, especially in precision, where we outperform the second highest, Densenet, by more than 10*%*; second, for the f1 score, which can take into account both positive and negative samples, our method also outperforms the rest of the networks by about 5*%*; finally, for our main metric, AUC, we also surpass the other networks by a large margin. As for the classification of seborrheic keratosis, although the advantage of our method is not as obvious as when classifying melanoma, it still performs well. First, our method still outperforms the other networks in terms of AUC, which is our main metric; second, for precision and ACC, our method leads by a small margin; and for recall and f1, we are slightly below the performance of Inception-Resnet-v2 and Xception. In general, our method is very efficient for classifying melanoma, although it is not significantly superior for classifying seborrheic keratosis, so it can improve the accuracy of classification in this task in general.

**FIGURE 8:**
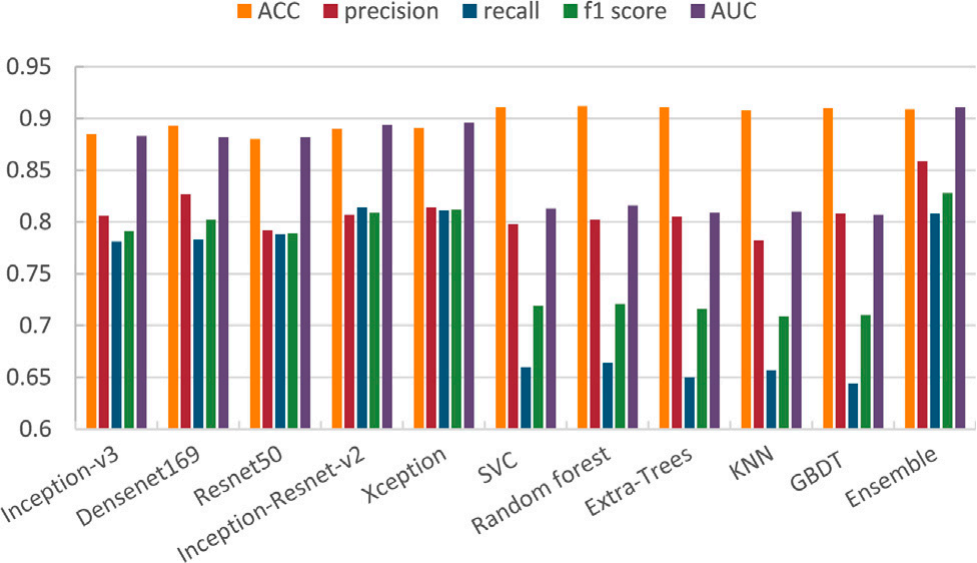
Comparison of different methods on skin lesion classification.

We average the performance of all networks and ensemble methods on two binary tasks and show them in Table 5. When compared with a single network, it can be seen that our ensemble method can effectively improve the performance; especially the AUC is 1*%* better than the best single network, i.e., Xception. At the same time, for precision and f1 score, our ensemble network is also the highest one. In addition, when compared with other ensemble methods, we use several machine learning classifier to do ensemble as comparison. We can see that except that ACC is 0.003 lower than Random forest, we are significantly better than machine learning methods on other metrics. We also illustrate this comparison in Figure 8, so we can more intuitively see the advantages of our ensemble method in various metrics.

**TABLE 5:** Average results of two skin lesion classifications of different networks.

Methods	ACC	Precision	Recall	f1 score	AUC
Inception-v3	0.885	0.806	0.781	0.791	0.883
Densenet169	0.893	0.827	0.783	0.802	0.882
Resnet50	0.88	0.792	0.788	0.789	0.882
Inception-Resnet-v2	0.89	0.807	**0.814**	0.809	0.894
Xception	0.891	0.814	0.811	0.812	0.896
SVC^1^	0.911	0.798	0.66	0.719	0.813
Random forest	**0.912**	0.802	0.664	0.721	0.816
Extra-Trees	0.911	0.805	0.65	0.716	0.809
KNN	0.908	0.782	0.657	0.709	0.81
GBDT^2^	0.91	0.808	0.644	0.71	0.807
Ensemble	0.909	**0.859**	0.808	**0.828**	**0.911**

1 Support Vector Classification.

2 Gradient Boost Decision Tree.

### 4.6 Comparison of Various Predictors

In Table 6, we compare our method with the top five performance in the ISIC 2017 challenge skin lesion classification task (Díaz, 2017; Matsunaga et al., 2017; Bi et al., 2017; Menegola et al., 2017; Yang et al., 2017) and some excellent methods in recent years. Most of the networks participating in the challenge used external images, which we do not do. In Table 6, it can be seen that our method achieves 0.909 and 0.859 on ACC and precision, which are highest on these metrics. Besides, we get 0.911 on AUC, which is 0.048 lower than that of Datta et al. (2021). For f1 score, our method obtains 0.828, which is 0.023 lower than the best score. However, for recall, our model’s performance is a bit unsatisfactory, which shows that our model still has some shortcomings in classifying positive samples.

**TABLE 6:** Comparison among our method, some existing methods, and the top five ISIC2017 classification challenge.

Method	ACC	Precision	Recall	f1 score	AUC
Top 1	0.816	0.748	0.856	**0.851**	0.911
Top 2	0.849	0.747	0.140	0.236	0.910
Top 3	0.883	0.752	0.451	0.564	0.908
Top 4	0.888	0.732	0.508	0.600	0.896
Top 5	0.873	0.665	0.568	0.613	0.886
Zhang et al. (2019)	0.868	—	0.878	—	0.958
González-Díaz (2019)	—	—	—	—	0.917
Xie et al. (2020)	0.904	—	0.786	—	0.938
Datta et al. (2021)	0.833	—	**0.916**	—	**0.959**
Ours	**0.909**	**0.859**	0.808	0.828	0.911

## 5 Conclusion

In this paper, we have the following innovations: 1) we propose a new two-stage ensemble method that integrates five excellent classification models to classify skin melanoma; 2) we also propose a new method of segmenting the lesion area of the dermoscopy image to generate a mask of the lesion area, so that the image can be resized to focus on the lesion; 3) we propose a new ensemble network that can use local connected layers to effectively integrate the classification results from the five classification networks. We test our method on the ISIC 2017 challenge dataset and get pretty good results. In future work, we will explore more effective classification methods based on the characteristics of dermoscopy images and the association of different classes of dermoscopy images, especially in process of pre-processing, because the experimental results show that our segmented images can largely improve the accuracy of classification.

## Data Availability

The original contributions presented in the study can be accessed at https://github.com/guofei-tju/Melanoma_cls. Further inquiries can be directed to the corresponding authors.
